# Research on the Physiological Response Mechanism and Expression of Key Leaf Color Genes in ‘Duojiao’ Crabapple Under Partial Shading

**DOI:** 10.3390/plants15101552

**Published:** 2026-05-19

**Authors:** Bingyuan Chen, Min Wang, Yuhan Yang, Luoya Li, Yuwei Fan, Xiajing Zong, Xiaoqian Guo, Feiran Zou, Qiankun Lin, Hongyan Yu, Jianlong Yu, Manman Zhang, Yunfei Mao, Xiang Shen

**Affiliations:** 1 State Key Laboratory of Crop Biology, College of Horticulture Science and Engineering, Shandong Agricultural University, Tai’an 271002, China; bing19992025@163.com (B.C.); 13562568381@163.com (M.W.); 18035154796@163.com (Y.Y.); 19550902896@163.com (L.L.); 13665336694@163.com (Y.F.); 18864803677@163.com (X.Z.); 13808998638@163.com (X.G.); zfr0718@outlook.com (F.Z.); zhangmanman@sdau.edu.cn (M.Z.); 2 Yantai Zhongquan Food Co., Ltd., Yantai 265307, China; 15653585567@163.com; 3 Shenyang Meijiaou Ornamental Tree Species R&D Co., Ltd., Shenyang 110121, China; sykxkj@163.com (H.Y.); lnmjoyl@163.com (J.Y.)

**Keywords:** ‘Duojiao’ crabapple, partial shading, leaf color regulation, chlorophyll metabolism, gene function validation

## Abstract

The first yellow-leafed crabapple variety developed in China is *Malus* ‘Duojiao’. The light level affects its leaf color. (1) Background: Plants are frequently shaded by photovoltaic panels and green buildings. It is unknown how genetic regulation and partial shadowing regulate leaf color. (2) Methods: Four 28-day shading treatments were used for ‘Duojiao’ crabapple and its maternal ‘Xifu’ crabapple. Virus-induced gene silencing (VIGS), overexpression transgenic validation experiments, and physiological index analysis were employed to identify the expression levels of significant candidate genes. (3) Results: Improvements in chlorophyll synthesis, mineral metabolism, and antioxidant status were observed. The net photosynthetic rate was 39.29% higher under double-layer shade than in the control. (4) Conclusions: Partial double-layer shading exhibited the optimal effect. *MsCPOX* was the key gene controlling leaf color. Our results provide a theoretical basis for analyzing light responses and determining genes regulating leaf color in crabapple.

## 1. Introduction

Crabapple (*Malus* spp.), an attractive species in the Rosaceae family, is native to China and has been cultivated for more than 2000 years [1]. There are more than a thousand cultivars worldwide. Crabapple is an important landscape tree species and is prized for its flowers, leaves, and fruits. This species has abundant genetic resources [2]. The first yellow-leaf ornamental crabapple variety to be independently developed in China and registered worldwide is *Malus* ‘Duojiao’. It was derived from a bud sport of the maternal parent *Malus* ‘Xifu’. This variety has a unique leaf color, transitioning from yellow to green, providing exceptional ornamental value and market potential [3].

China is promoting the synergistic development of photovoltaic agriculture and green architecture [4]. Light affects plant leaf color, photosynthetic efficiency, and photosynthetic pigment metabolism [5]. However, most studies on crabapple shade tolerance have been empirical, and systematic research on this species’ physiological adaptation and molecular regulation under partial shade is lacking [6]. However, current assessments of crabapple shade tolerance are largely empirical, lacking systematic research on physiological adaptation and molecular regulation under partial shade [7], limiting crabapple cultivar breeding in shaded habitats [8].

Leaf color is a core ornamental trait of crabapples. Light affects pigment metabolism through gene expression, altering leaf color [9]. Most studies have focused on the general effect of shading on plants [10]. In contrast, few studies have examined the physiological responses and gene expression at the leaf level under partial shading. This information is required to elucidate how dappled light influences plant adaptation and leaf color [11,12]. Genes related to chlorophyll metabolism are key targets for leaf color regulation [13]. *MsCAO*, *MsCPOX*, and *MsGLK1* are regulated by light and participate in chlorophyll synthesis, metabolism, and chloroplast development, respectively [14,15,16]. They are core candidate genes for regulating leaf color in ‘Duojiao’ crabapple; however, their regulatory mechanisms remain unclear.

Potential genes were selected by our team using transcriptome analysis. Preliminary research indicates that a shade treatment resulted in significant leaf color changes and an increased chlorophyll content in the ‘Duojiao’ variety. However, the physiological responses under local shading, the validation of gene expression via transgenesis, and independent leaf responses remain to be verified. Based on the theoretical foundation and previous findings, the following hypotheses are proposed: (1) Local shading induces independent and reversible physiological adaptations in the leaves of *Malus* ‘Duojiao’ by increasing chlorophyll content and optimizing the photosynthetic pigment ratio. The single-leaf response is independent of whole-plant responses. (2) Local shading significantly upregulates the expression levels of *MsCAO*, *MsCPOX*, and *MsGLK1*, promoting chlorophyll synthesis and chloroplast development, and mediating the transition of yellow to green leaves. This study examines the effect of local shading on *Malus* ‘Duojiao’ and systematically investigates the physiological and molecular responses underlying leaf color regulation. The aim is to fill the research gap on responses to local light intensity and to provide theoretical support and a scientific basis for the role of *Malus* ‘Duojiao’ as a landscape species, for breeding of shade-tolerant varieties, and for molecular genetic improvement.

## 2. Results and Analysis

### 2.1. Effects of Partial Shading Treatment on Physiological Characteristics of Crabapple

#### 2.1.1. Effects on Photosynthetic and Fluorescence Characteristics

**Figure 1:**
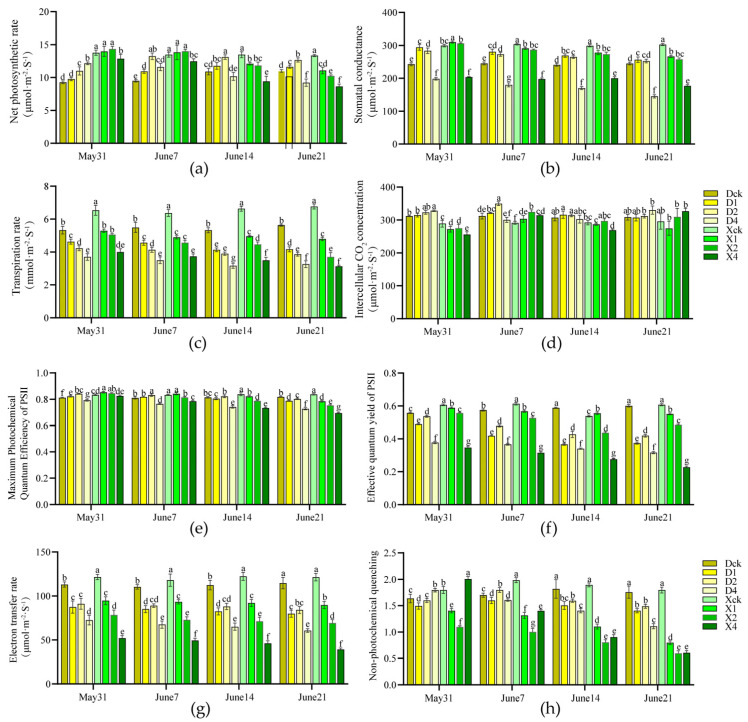
Effects of partial shading treatment on the photosynthetic (**a**–**d**) fluorescence (**b**–**h**) characteristics of crabapple leaves (*p* < 0.05). Note: Dck represents the control treatment of *Malus* ‘Duojiao’ leaves, D1 represents the single-layer shade treatment of *Malus* ‘Duojiao’ leaves, D2 represents the double-layer shade treatment of *Malus* ‘Duojiao’ leaves, and D4 represents the four-layer shade treatment of *Malus* ‘Duojiao’ leaves. Xck represents the control treatment of *Malus* ‘Xifu’ leaves, X1 represents the single-layer shade treatment of *Malus* ‘Xifu’ leaves, X2 represents the double-layer shade treatment of *Malus* ‘Xifu’ leaves, and X4 represents the four-layer shade treatment of *Malus* ‘Xifu’ leaves. (**a**) The figure illustrates how local shading treatment affects the net photosynthetic rate of crabapple leaves. (**b**) The effect of local shading treatment on the stomatal conductance of crabapple leaves. (**c**) The effect of local shading treatment on the transpiration rate of crabapple leaves. (**d**) The figure illustrates how the intercellular CO_2_ content of crabapple leaves is affected by local shading treatment. (**e**) The figure illustrates how local shade treatment affects crabapple leaves’ Maximum photochemical quantum yield of PSII. (**f**) The figure illustrates how local shading treatment affects crabapple leaves’ Effective Quantum yield of PSII. (**g**) The figure illustrates how the electron transport rate of crabapple leaves is affected by local shading treatment. (**h**) The figure illustrates how local shade treatment affects crabapple leaves’ non-photochemical quenching. Different lowercase letters in the figure indicate significant differences between samples at different time points (*p* < 0.05; one-way ANOVA).

Partial shading significantly affected the photosynthetic characteristics of ‘Duojiao’ and ‘Xifu’ crabapple (*p* < 0.05) (Figure 1a–d). The net photosynthetic rate of ‘Duojiao’ crabapple increased and decreased, peaking at 13.233 μmol·m^−2^·s^−1^ on day 14 under double-layer shade (D2, 30–35%). The photosynthetic rate was 39.29% higher than that of the control, indicating optimal photosynthetic efficiency. The photosynthetic rate of ‘Xifu’ crabapple increased transiently under single and double-layer shade but decreased significantly in later stages and under four-layer shade, demonstrating lower shade tolerance than ‘Duojiao’. Stomatal conductance for both species peaked on day 7. Stomatal conductance and transpiration rate were considerably lower under four-layer shade, although intercellular CO_2_ concentration increased with shading intensity.

The maximum photochemical quantum yield of PSII (Fv/Fm) for both cultivars exhibited minor changes with increasing chlorophyll fluorescence (Figure 1b–h) and reached the maximum on day 7. There was no discernible photodamage to PSII, with a value of 0.842 for the D2 group of ‘Duojiao’ 0.842 (3.65% higher than the control) and a value of 0.853 for the X1 group of ‘Xifu’ 0.853 (2.20% higher than the control). As the shade intensity and treatment time increased, the effective quantum yield of PSII (*Φ*_PSII_) and the electron transport rate (ETR) declined, with all treatments having lower values than the control on day 28. Non-photochemical quenching (NPQ) increased during the initial stage, with ‘Duojiao’ exhibiting higher values than ‘Xifu’ under long-term shading, suggesting superior photoprotective capacity. In general, four-layer shade considerably reduced photochemical efficiency, whereas single and double-layer shade had negligible effects on PSII function. The ‘Duojiao’ crabapple showed superior low-light tolerance.

#### 2.1.2. Effects on Mineral Element Content

**Figure 2:**
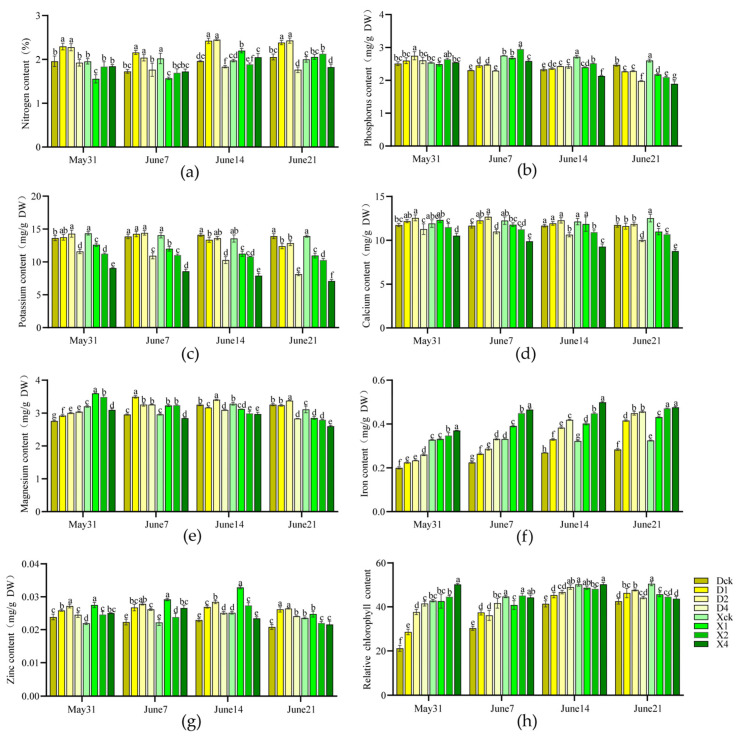
Effect of partial shading on mineral elements (**a**–**g**) and relative chlorophyll content in *Malus* crabapple leaves (**h**) (*p* < 0.05). Note: Dck represents the control treatment of Malus ‘Duojiao’ leaves, D1 represents the single-layer shade treatment of Malus ‘Duojiao’ leaves, D2 represents the double-layer shade treatment of Malus ‘Duojiao’ leaves, and D4 represents the four-layer shade treatment of Malus ‘Duojiao’ leaves. Xck represents the control treatment of Malus ‘Xifu’ leaves, X1 represents the single-layer shade treatment of Malus ‘Xifu’ leaves, X2 represents the double-layer shade treatment of Malus ‘Xifu’ leaves, and X4 represents the four-layer shade treatment of Malus ‘Xifu’ leaves. Different lowercase letters in the figure indicate significant differences between samples at different time points (*p* < 0.05; one-way ANOVA). (**a**) The figure shows the effect of local shading treatment on the nitrogen content of crabapple leaves. (**b**) The figure shows the effect of local shading treatment on the phosphorus content of crabapple leaves. (**c**) The figure shows the effect of local shading treatment on the potassium content of crabapple leaves. (**d**) The figure shows the effect of local shading treatment on the calcium content of crabapple leaves. (**e**) The figure shows the effect of local shading treatment on the magnesium content of crabapple leaves. (**f**) The figure shows the effect of local shading treatment on the iron content of crabapple leaves. (**g**) The figure shows the effect of local shading treatment on the zinc content of crabapple leaves. (**h**) The figure shows the effect of local shading treatment on the relative chlorophyll content of crabapple leaves.

Regarding macronutrients, ‘Duojiao’ crabapple’s nitrogen, phosphorus, and potassium concentrations were metabolically stable under single and double-layer shade and slightly lower under four-layer shade. In contrast, the macronutrients of ‘Xifu’ crabapple were more sensitive to shading. As the shading intensity increased, the contents of nitrogen, phosphorus, and potassium decreased considerably, suggesting impeded nutrient accumulation. Regarding micronutrients, the ‘Duojiao’ crabapple’s calcium and magnesium contents remained high under double-layer shade, whereas its iron and zinc concentrations increased slightly with shading intensity, indicating a stable metabolism. Most micronutrients of ‘Xifu’ crabapple increased and decreased. Their contents were significantly lower than those of ‘Duojiao’ under long-term shading, confirming the superior shade tolerance of ‘Duojiao’ crabapple (Figure 2a–g).

#### 2.1.3. Effects on Chlorophyll Content

Partial shading significantly increased chlorophyll content in both cultivars, with different responses across varieties. The relative chlorophyll content of ‘Duojiao’ crabapple was higher than that of the control in all shading treatments. It increased with treatment duration, peaking at 28 days. The D2 treatment group showed the greatest effect, with a relative chlorophyll content of 47.667, which was 12.07% higher than the control, indicating that ‘Duojiao’ accumulated more chlorophyll, even under prolonged low-light conditions. In contrast, the chlorophyll content of ‘Xifu’ crabapple increased initially, peaked at 21 days, and declined. By day 28, the chlorophyll content was lower in all treatments than in the control, suggesting that only short-term moderate shading slightly improved chlorophyll synthesis in ‘Xifu’, whereas long-term shading had a significant inhibitory effect (Figure 2h).

**Figure 3:**
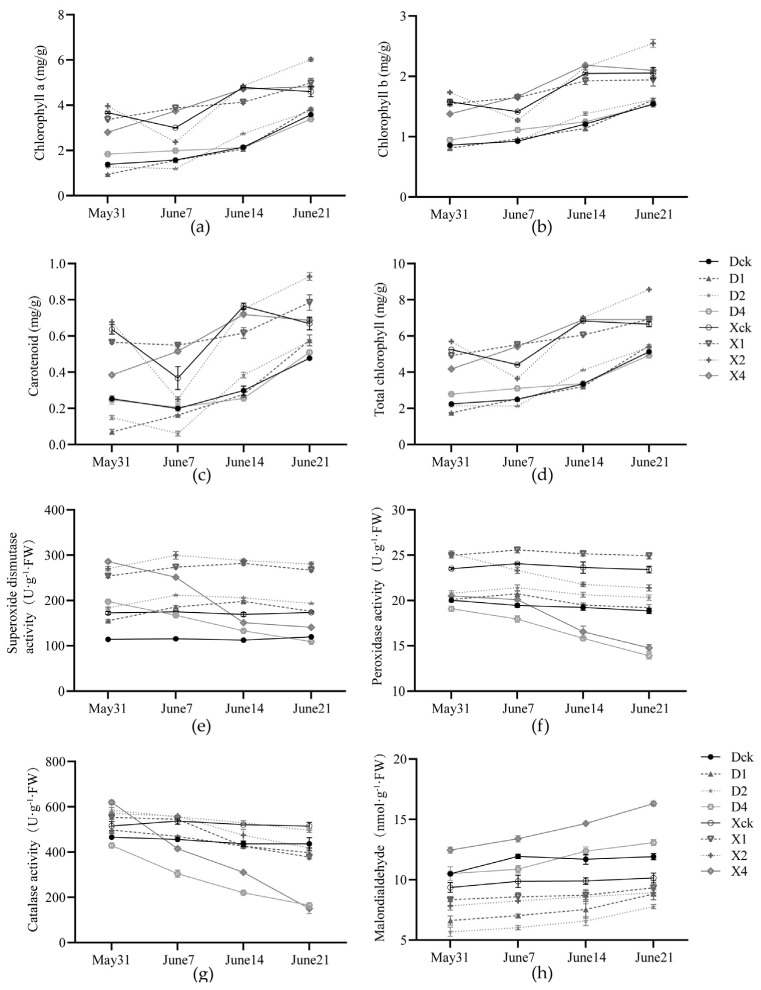
Effect of local shading on chlorophyll content (**a**–**d**) in crabapple leaves and the systemic response of resistance enzymes (**e**–**h**) (*p* < 0.05). Note: Dck represents the control treatment of Malus ‘Duojiao’ leaves, D1 represents the single-layer shade treatment of Malus ‘Duojiao’ leaves, D2 represents the double-layer shade treatment of Malus ‘Duojiao’ leaves, and D4 represents the four-layer shade treatment of Malus ‘Duojiao’ leaves. Xck represents the control treatment of Malus ‘Xifu’ leaves, X1 represents the single-layer shade treatment of Malus ‘Xifu’ leaves, X2 represents the double-layer shade treatment of Malus ‘Xifu’ leaves, and X4 represents the four-layer shade treatment of Malus ‘Xifu’ leaves.

The ‘Duojiao’ crabapple’s carotenoid, total chlorophyll, chlorophyll a, and chlorophyll b concentrations increased with shading duration, peaking at 28 days. All treatments outperformed the control, with the D1 treatment showing the highest levels of carotenoid and chlorophyll a, and the D2 treatment group showing the highest levels of chlorophyll b and total chlorophyll. The chlorophyll indices of ‘Xifu’ crabapple were higher than those of ‘Duojiao’, reaching their maximum in the X2 treatment group. The total chlorophyll content was 8.570 mg·g^−1^, which was 28.79% higher than that of the control. Double-layer shading had the most significant positive effect on chlorophyll synthesis in both cultivars (Figure 3a–d).

#### 2.1.4. Effects on the Antioxidant System and Key Enzymes in Chlorophyll Metabolism

Both cultivars’ antioxidant enzyme activity increased and decreased under partial shading (Figure 3e–h). Superoxide dismutase, peroxidase, and catalase activity in ‘Duojiao’ crabapple increased gradually and persisted longer in the D1 and D2 treatment groups, peaking at 14 days in the D2 group. The peak values in the treatments were 83.53%, 10.12%, and 25.52% higher, respectively, than in the control. The malondialdehyde content remained low, indicating stable regulation of the antioxidant system. Enzyme activities in the ‘Xifu’ crabapple only rose in the early stages under moderate shade but were substantially lower in the latter stages. The malondialdehyde level was significantly higher under four-layer shade, indicating significant membrane lipid peroxidation damage and low-light adaptation.

These results demonstrate that the enzyme activity in chlorophyll metabolism differed across cultivars (Table 1). As shading intensity and duration increased, plant δ- and glutamyl-tRNA reductase activity increased while aminolevulinic acid dehydratase activity decreased considerably. For both cultivars, the greatest inhibition was observed under four-layer shade. In general, ‘Xifu’ had higher enzyme activity than ‘Duojiao’. Uroporphyrinogen III synthase activity was consistently lower in ‘Duojiao’ than in ‘Xifu’. It exhibited an early increase followed by inhibition in ‘Duojiao’ but remained high, even under four-layer shading. In ‘Duojiao’ crabapple, the levels of chlorophyllase and Plant Mg-dechelatase activities were lower in all shading treatments than in the control. The activities declined with increasing shading intensity, suggesting slower chlorophyll breakdown. In contrast, the enzyme activity of ‘Xifu’ crabapple increased substantially with shading intensity, accelerating chlorophyll breakdown. The two cultivars exhibited opposite responses.

#### 2.1.5. Effects on the Expression of Key Leaf Color Genes

**Figure 4:**
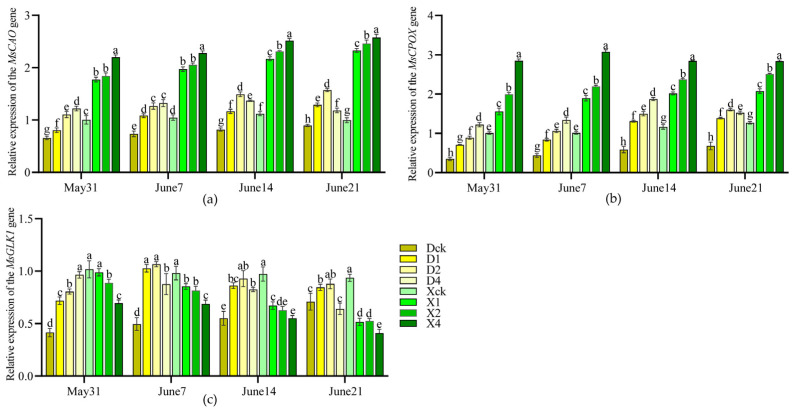
Analysis of the expression of key leaf color genes in ‘Duojiao’ under partial shading treatment (*p* < 0.05). Note: Dck represents the control treatment of Malus ‘Duojiao’ leaves, D1 represents the single-layer shade treatment of Malus ‘Duojiao’ leaves, D2 represents the double-layer shade treatment of Malus ‘Duojiao’ leaves, and D4 represents the four-layer shade treatment of Malus ‘Duojiao’ leaves. Xck represents the control treatment of Malus ‘Xifu’ leaves, X1 represents the single-layer shade treatment of Malus ‘Xifu’ leaves, X2 represents the double-layer shade treatment of Malus ‘Xifu’ leaves, and X4 represents the four-layer shade treatment of Malus ‘Xifu’ leaves. Different lowercase letters in the figure indicate significant differences between samples at different time points (*p* < 0.05; one-way ANOVA). (**a**) The figure shows the effect of local shading treatment on the relative expression of the candidate key gene *MsCAO* for leaf color in ‘Duojiao’ crabapple. (**b**) The figure shows the effect of local shading treatment on the relative expression of the candidate key gene *MsCPOX* for leaf color in ‘Duojiao’ crabapple. (**c**) The figure shows the effect of local shading treatment on the relative expression of the candidate key gene *MsGLK1* for leaf color in ‘Duojiao’ crabapple.

Partial shading independently regulated the expressions of *MsCAO*, *MsCPOX*, and *MsGLK1* genes in leaves, with significant differences in expression patterns between the two cultivars (*p* < 0.05) (Figure 4). As treatment duration increased, the expression levels of *MsCAO* and *MsCPOX* initially increased, then decreased. The expression levels of both genes were higher in the single- and double-layer shade treatments than in the control, with *MsCAO* upregulation greater in ‘Xifu’ than in ‘Duojiao’ crabapple. The D2 treatment group of the ‘Duojiao’ crabapple exhibited the optimal expression. After 28 days, the expression levels of *MsCAO*, *MsCPOX*, and *MsGLK1* were 1.756, 2.354, and 1.11–2.16 times that of the control, respectively. In contrast, the expression level of *MsGLK1* in ‘Xifu’ crabapple was lower than that of the control. Four-layer shade inhibited gene expression. These findings demonstrate that the ‘Duojiao’ crabapple has more pronounced shade tolerance and that the leaf’s response to shading is not subject to whole-plant regulation, which is consistent with the observed changes in physiological indicators and leaf color.

### 2.2. Cloning and Bioinformatics Analysis of Key Leaf Color Genes

Homologous cloning was used to determine the coding sequences of the *MsCAO* (LOC103431681), *MsCPOX* (LOC103408418), and *MsGLK1* (LOC103444434) genes from the ‘Duojiao’ crabapple. The base pair lengths were 1587, 1636, and 1534, respectively. The target fragments for the virus-induced gene silencing (VIGS) vectors were cloned. Sequence alignment showed that the similarity of the three genes to homologous genes in *Malus* species from the NCBI database ranged from 95.45% to 96.17%. The similarity of the VIGS target fragments ranged from 87.31% to 93.05% (Figure S1), indicating their suitability for subsequent transgenic functional validation.

The bootstrap values exceeded 85% (Figure S2). Phylogenetic tree analysis showed that the MsCAO, MsCPOX, and MsGLK1 proteins clustered with those from other Rosaceae species, such as apple and pear, indicating evolutionary conservation. MsCAO and MsCPOX are basic, hydrophilic, stable proteins, whereas MsGLK1 is a neutral, hydrophilic, unstable protein. An analysis of the physicochemical characteristics showed that 70% of the secondary structures were alpha helices and random coils (Table S1). Transmembrane region and tertiary structure predictions indicated that MsCAO and MsCPOX contained transmembrane helices (Figure S3), characteristic of membrane-bound enzymes, whereas MsGLK1 lacked transmembrane regions and had a DNA-binding domain (Figure S4). These structural characteristics are highly consistent with their functional roles.

### 2.3. Functional Validation of Key Leaf Color Genes

#### 2.3.1. Effects of VIGS Silencing on Plant Crabapple and Chlorophyll Content

**Figure 5:**
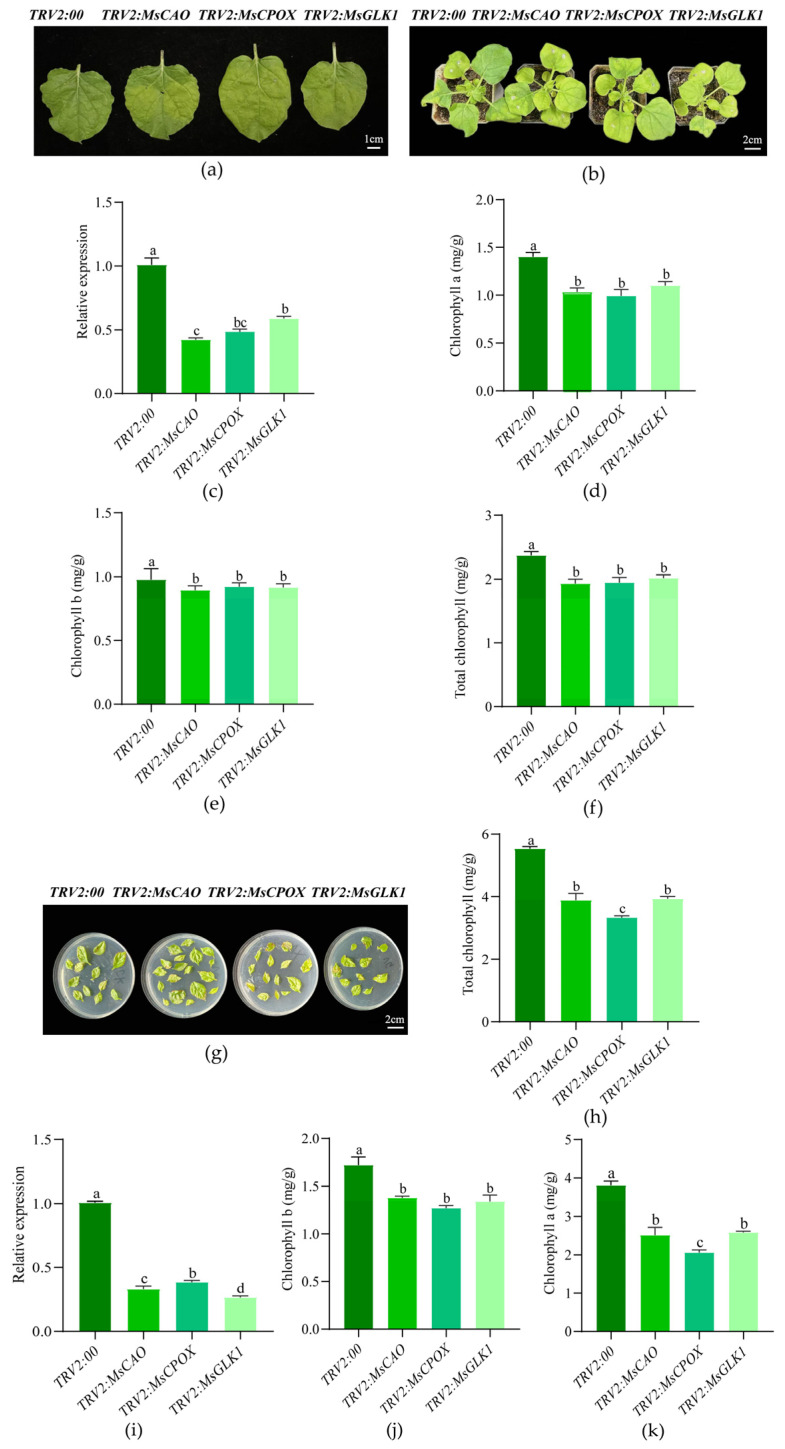
Phenotypic and chlorophyll content changes in Virus-induced gene silencing tobacco (**a**–**f**) and Gala apples plants (**g**–**k**) for key candidate genes controlling the leaf color of ‘Duojiao’ crabapple. Note: TRV2:00 represents the control treatment for virus-induced gene silencing, *TRV2:MsCAO* represents the virus-induced silencing of the *MsCAO* gene, *TRV2:MsCPOX* represents the virus-induced silencing of the *MsCPOX* gene, and *TRV2:MsGLK1* represents the virus-induced silencing of the *MsGLK1* gene. Different lowercase letters in the figure indicate significant differences between samples at different time points (*p* < 0.05; one-way ANOVA). (**a**) Figure showing tobacco leaves 5 days after virus-induced silencing of the key gene regulating ‘Duojiao’ crabapple leaf color. Scale as shown in the figure. (**b**) Figure showing tobacco plants 7 days after virus-induced silencing of the key gene regulating ‘Duojiao’ crabapple leaf color. Scale as shown in the figure. (**c**) Figure showing the relative expression levels of genes in tobacco leaves 5 days after virus-induced silencing of the key gene regulating ‘Duojiao’ crabapple leaf color. (**d**) Figure showing chlorophyll a content in tobacco leaves 5 days after virus-induced silencing of the key gene regulating ‘Duojiao’ crabapple leaf color. (**e**) Figure showing chlorophyll b content in tobacco leaves 5 days after virus-induced silencing of the key gene regulating ‘Duojiao’ crabapple leaf color. (**f**) Figure showing total chlorophyll content in tobacco leaves 5 days after virus-induced silencing of the key gene regulating ‘Duojiao’ crabapple leaf color. (**g**) Figure showing Gala leaves 5 days after virus-induced silencing of the key gene regulating ‘Duojiao’ crabapple leaf color. Scale as shown in the figure. (**h**) Figure showing total chlorophyll content in Gala leaves 5 days after virus-induced silencing of the key gene regulating ‘Duojiao’ crabapple leaf color. (**i**) Figure showing the relative expression levels of genes in Gala leaves 5 days after virus-induced silencing of the key gene regulating ‘Duojiao’ crabapple leaf color. (**j**) Figure showing chlorophyll b content in Gala leaves 5 days after virus-induced silencing of the key gene regulating ‘Duojiao’ crabapple leaf color. (**k**) Figure showing chlorophyll a content in Gala leaves 5 days after virus-induced silencing of the key gene regulating ‘Duojiao’ crabapple leaf color.

Following transformation with *TRV2:MsCAO*, *TRV2:MsCPOX*, and *TRV2:MsGLK1* vectors, the VIGS gene silencing experiments demonstrated (Figure 5) that target gene expression levels were downregulated to 42–52% of the control in tobacco (Figure 5a–f) and to 26–38% of the control in Gala apples (Figure 5g–k), indicating a successful silencing system. Phenotypic observations showed that the leaves of all silenced plants exhibited chlorosis, with the *TRV2:MsCPOX* treatment showing the most pronounced chlorosis and the lowest chlorophyll content. The *TRV2:MsCAO* group’s chlorophyll concentration in tobacco was 18.77% lower than that of the control. The *TRV2:MsCPOX* group’s chlorophyll a level in Gala apples was 2.21 mg·g^−1^ lower than the control group’s. The results indicate that *MsCAO* and *MsCPOX* are key genes regulating chlorophyll synthesis, with the latter having greater importance, whereas *MsGLK1* had a smaller effect on color expression.

#### 2.3.2. Effects of Overexpression on *Arabidopsis* Crabapple and Chlorophyll Content

**Figure 6:**
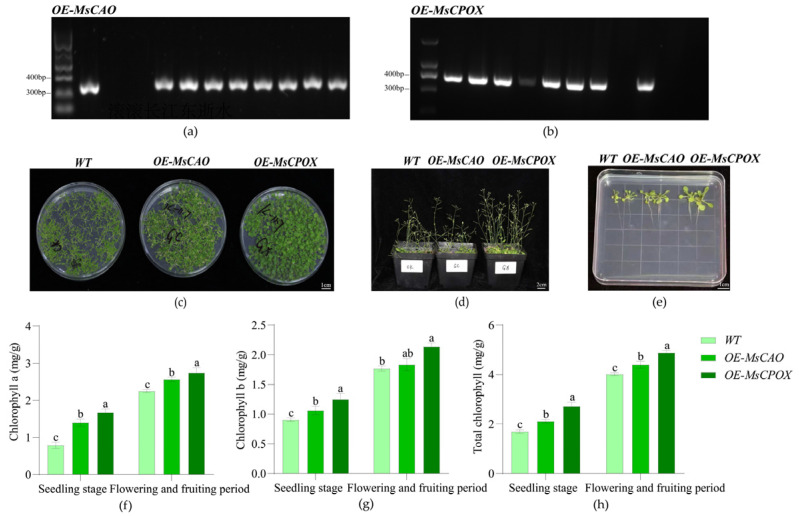
Crabapple of ‘Duojiao’ crabapple candidate leaf color gene overexpression plants and changes in chlorophyll content. Note: Different lowercase letters in the figure indicate significant differences between samples at different time points (*p* < 0.05; one-way ANOVA). (**a**) The figure shows the agarose gel electrophoresis of *Arabidopsis* overexpressing the key candidate gene *MsCAO* for ‘Duojiao’ crabapple leaf color. *OE-MsCAO* spotting order: marker, wild-type control, water control, lines 1–8. (**b**) The figure shows the agarose gel electrophoresis of *Arabidopsis* overexpressing the key candidate gene *MsCPOX* for ‘Duojiao’ crabapple leaf color. *OE-MsCPOX* spotting order: marker, lines 1–9, wild-type control, water control. (**c**) The figure shows the seedling stage images of *Arabidopsis* overexpressing the key candidate gene for ‘Duojiao’ crabapple leaf color. Scale as shown in the figure. (**d**) The figure shows the flowering and fruiting stage images of *Arabidopsis* overexpressing the candidate gene for ‘Duojiao’ crabapple leaf color. Scale as shown in the figure. (**e**) The figure shows individual seedling images of *Arabidopsis* overexpressing the key candidate gene for ‘Duojiao’ crabapple leaf color. Scale as shown in the figure. (**f**) The figure shows the chlorophyll a content in seedlings and the flowering/fruiting stage of *Arabidopsis* overexpressing the key candidate gene for ‘Duojiao’ crabapple leaf color. (**g**) The figure shows the chlorophyll b content in seedlings and flowering/fruiting stage of *Arabidopsis* overexpressing the key candidate gene for ‘Duojiao’ crabapple leaf color. (**h**) The figure shows the total chlorophyll content in seedlings and the flowering/fruiting stage of *Arabidopsis* overexpressing the key candidate gene for ‘Duojiao’ crabapple leaf color.

Positive transgenic lines overexpressing *OE-MsCAO* and *OE-MsCPOX* were developed in the *Arabidopsis* overexpression experiment (Figure 6). The leaves’ chlorophyll content changed noticeably. The overexpression lines exhibited better growth than the wild type, with a growth vigor ranking of *OE-MsCPOX* > *OE-MsCAO* > wild type. Larger leaves and copious bolting were signs of more aggressive growth in plants overexpressing *OE-MsCPOX*. The overexpression lines of both genes had considerably higher chlorophyll contents than the wild type. The chlorophyll content of the *MsCPOX* overexpression lines was 60.27% and 21.57% higher, respectively, than that of the wild type, with values of 2.71 mg·g^−1^ at the seedling stage and 4.22 mg·g^−1^ at the blooming and fruiting stage. This finding demonstrates that increasing photosynthetic efficiency by *OE-MsCPOX* affects chlorophyll synthesis and improves plant growth and development.

## 3. Discussion

The dominant factor controlling plant physiology and leaf color is light [17]. Most research has focused on the plant response to shading [18,19,20], whereas systematic studies on the physiological adaptation, molecular regulation, and response independence at the leaf level under partial shade are lacking [21,22]. The regulation of plant leaf color under partial shading is difficult to analyze [23]. This study examined the effect of partial shade on the color expression of the yellow-leaf ornamental crabapple variety ‘Duojiao’. The findings are highly consistent with theories on shade tolerance in colored-leaf plants and on chlorophyll metabolism regulation [24] while addressing a knowledge gap regarding responses to partial shading [25].

Numerous studies have demonstrated that moderate shade can improve the physiology of sun-loving plants and alleviate light inhibition [26,27,28,29]. Most ornamental crabapples, such as *Prunus cerasifera* Pissardii, and other colored-leaf plants benefit from 30% to 40% shade because it improves light-use efficiency, maintains photosynthetic stability, and prevents a decrease in photosynthetic efficiency due to shade [30,31,32,33,34,35]. The ‘Duojiao’ crabapple’s markedly increased photosynthetic efficiency and stable PSII system under 30–35% double-layer shade in this study is consistent with these findings, demonstrating the beneficial effect of moderate shading on plant photosynthetic physiology [36]. Even under prolonged shading, the ‘Duojiao’ crabapple exhibited higher photosynthetic efficiency and photoprotective capacity than the green leaf ‘Xifu’ crabapple, displaying noticeably larger NPQ values. These results are consistent with hypothesis 1. This finding demonstrates the low-light adaptability of the yellow-leaf ‘Duojiao’ cultivar and is consistent with the adaptation mechanism of shade-tolerant plants, which use thermal dissipation to prevent damage to the photosynthetic system and preserve pigment stability [37].

A balanced chlorophyll metabolism is required for regulating plant leaf color [38]. The leaf color of plants growing in the shade changes due to pigment accumulation and the optimization of pigment composition. It is controlled by enzymes involved in chlorophyll synthesis and degradation [39]. Shade-tolerant plants can capture light under low-light conditions by increasing their chlorophyll b content [40] while slowing chlorophyll breakdown by blocking the actions of degradative enzymes, such as Mg-dechelatase and chlorophyllase, creating a metabolic equilibrium characterized by high synthesis and low degradation [41]. The significant increase in chlorophyll content and the sustained inhibition of degradative enzyme activities in ‘Duojiao’ crabapple under partial shading observed in this study are consistent with shade tolerance [42]. In contrast, the ‘Xifu’ crabapple exhibited an unstable metabolic pattern of high synthesis and high degradation, consistent with the shading response of most green-leaf plants. Mineral nutrients and the antioxidant system are crucial for chlorophyll metabolism [43]. Recent studies have shown that consistent antioxidant enzyme activity and a sufficient supply of metals like iron and magnesium maintain chloroplast structure and sustain pigment formation [44]. The results of this study are highly consistent with previous investigations.

Light affects leaf coloration by molecular regulation of gene expression [45]. Genes such as *CAO*, *CPOX*, and *GLK1* are core regulators of chlorophyll metabolism and chloroplast development. Light intensity affects their expression patterns [46,47,48]. *GLK1* is a crucial transcription factor that controls photosynthetic gene expression and chloroplast growth, which are essential for chlorophyll accumulation [49]. The effectiveness of chlorophyll accumulation is directly determined by the expression level of *CPOX*, which codes for a rate-limiting enzyme in the chlorophyll production pathway [50,51]. By converting chlorophyll a to chlorophyll b, CAO optimizes the composition of pigments [52]. In this study, double-layer shading significantly increased the expression levels of these three genes in ‘Duojiao’ crabapple while inhibiting GLK1 expression in ‘Xifu’ crabapple. These results are consistent with hypothesis 2 and align with other studies on the molecular control of leaf color in colored-leaf plants.

The most novel finding of this study is that the leaves of the ‘Duojiao’ crabapple responded independently to partial shading. This finding contradicts conventional knowledge of whole-plant systemic regulation. Few studies have investigated the responses of individual leaves to light. In line with findings from studies on local stress responses in forest trees and herbaceous plants, this study found that the shade effect was limited to leaves and did not propagate through systemic signals [53]. This finding provides a theoretical basis for the targeted regulation of plant leaf color in dappled light environments.

This study has some limitations. The light level was not evaluated. No homologous transformation validation was performed, and the functional validation of genes was conducted on heterologous systems. Moreover, the interactions between fundamental genes were not analyzed. The local light responses of colored-leaf plants should be assessed in depth using gene interaction analysis and red-to-blue light ratios to provide a more detailed theoretical basis for breeding shade-tolerant ornamental crabapple varieties.

## 4. Materials and Methods

### 4.1. Experimental Materials

Twenty potted seedlings of three-year-old *Malus* ‘Duojiao’ and the maternal parent *Malus* ‘Xifu’, which were grafted onto *Malus* (Pingyi Tiancha) rootstocks and displaying sufficient growth vigor, were used. Tissue culture seedlings of Gala apples, *Nicotiana benthamiana*, and *Arabidopsis thaliana* were used for transgenic experiments. The photoperiod was 16 h of light and 8 h of darkness. The ambient temperature was 23 °C, and an MS solid medium was used for cultivation. All plants were grown under standard tissue culture and growth conditions to ensure consistency. The partial shading material was snow gauze black mesh. Single-, double-, and four-layer materials were utilized to create different shading levels. Bacterial strains (*Escherichia coli* DH5α, Trans1-T1, *Agrobacterium tumefaciens* competent cells GV3101 and LBA4404), vectors (cloning vector pEASY-blunt, overexpression vector pRI101, VIGS vectors pTRV1/pTRV2), and various ELISA kits (Jiangsu Meimian Industrial Co., Ltd., Yancheng, China) were used. Total RNA was extracted according to the instructions of the RNAprep Pure Plant Kit (Tiangen, China). Quantitative reverse transcription polymerase chain reaction (qRT-PCR) was performed using the SYBR Green Pro TaqHS pre-mixed fluorescent method developed by Hunan Aikorui Bioengineering Company (Changsha, China).

### 4.2. Experimental Design

#### 4.2.1. Partial Shading Treatment

**Figure 7:**
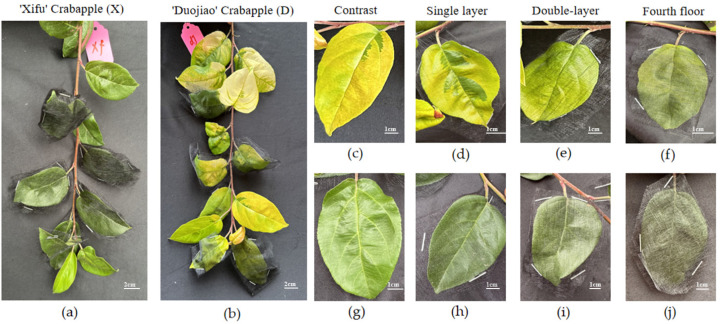
Comparison of leaves of ‘Duojiao’ Crabapple and ‘Xifu’ Crabapple under different local shading treatments for 14 days. Note: (**a**) The figure shows different local shading treatments on the entire branch of ‘Xifu’ crabapple. (**b**) The figure shows different local shading treatments on the entire branch of ‘Duojiao’ crabapple. (**c**) The figure shows the unshaded control of ‘Duojiao’ crabapple leaves. (**d**) The figure shows single-layer shading of ‘Duojiao’ crabapple leaves. (**e**) The figure shows double-layer shading of ‘Duojiao’ crabapple leaves. (**f**) The figure shows four-layer shading of ‘Duojiao’ crabapple leaves. (**g**) The figure shows the unshaded control of ‘Xifu’ crabapple leaves. (**h**) The figure shows single-layer shading of ‘Xifu’ crabapple leaves. (**i**) The figure shows double-layer shading of ‘Xifu’ crabapple leaves. (**j**) The figure shows four-layer shading of ‘Xifu’ crabapple leaves. Scale as shown in the figure.

Four shading levels were used in the experiment conducted in late May 2024: natural light (0% shade, CK), single-layer shade (15–20%), double-layer shade (30–35%), and four-layer shade (60–70%). Four sturdy new branches were chosen from the outer canopy of each plant, and leaves 5–13 on these shoots were partially shaded (Figure 7). Samples were collected at predetermined intervals every week during the four-week experiment to measure target gene expression levels and leaf physiological parameters. Five potted biological replicates were used for each treatment.

#### 4.2.2. Gene Functional Validation Experiment

The three key genes *MsCAO*, *MsCPOX*, and *MsGLK1* were cloned, and VIG vectors and overexpression vectors were constructed. *Agrobacterium*-mediated methods were used to conduct VIGS in *Nicotiana benthamiana* and Gala apples. Stable genetic transformation was performed using *Arabidopsis thaliana*. Gene functions were elucidated by observing crabapples and measuring physiological indices.

### 4.3. Experimental Methods

#### 4.3.1. Determination of Physiological Indices

Photosynthetic and fluorescence characteristics: A YLS-501 chlorophyll meter was used to measure soil–plant analysis development (SPAD) from 8:30 to 11:30 on sunny days. A Junior-PAM fluorometer was used to measure chlorophyll fluorescence parameters. A CIRAS-3 photosynthesis system (with 1200 µmol·m^−2^·s^−1^ LED light intensity) was used to measure gas exchange parameters, such as net photosynthetic rate and stomatal conductance [54].

Chlorophyll content: 0.1 g of fresh leaf samples were extracted in 95% ethanol in the dark until the tissue turned white [55]. Ultraviolet (UV) spectrophotometry was used to measure absorbance at 665 and 649 nm, and the contents of total chlorophyll, chlorophyll a, and chlorophyll b were determined.

Resistance indices and mineral elements: For fixation, the leaves were dried for 30 min at 105 °C and to a consistent weight at 80 °C. Following digestion with H_2_SO_4_-H_2_O_2_, the nitrogen content was determined using a Kjeldahl device (IShandong Haineng Scientific Instruments Co., Ltd., Jinan, China). The calcium content was determined using an atomic absorption spectrophotometer, and the phosphorus, potassium, magnesium, and other element concentrations were determined using inductively coupled plasma-optical emission spectrometry (ICP-OES). The guaiacol method, the nitrogen blue tetrazolium method, and other techniques were used to measure the activity of peroxidase, superoxide dismutase, and catalase, as well as the content of malondialdehyde [56]. The activities of key enzymes in chlorophyll metabolism were determined using ELISA kits.

#### 4.3.2. Molecular Biology Determinations

Reverse transcription and total RNA extraction: A specialist kit was used to extract total RNA from leaves. Following the removal of genomic DNA, complementary DNA was created using reverse transcription. It was kept at −20 °C for later use.

Gene cloning and vector construction: The company used complementary DNA as a template to synthesize gene primers (Table A1). The target gene was amplified using PCR and primers, and gel electrophoresis and sequencing were employed to retrieve the gel result. Homologous recombination and double restriction enzyme digestion of the vectors were used to select the restriction sites on the pTRV2 viral expression vector and the pRI101 empty vector using SnapGene (Version: 6.0, 2.0). The restriction sites chosen were SalI and BamHI for the F end and SmaI and KpnI for the R end. The FastDigest series of high-fidelity restriction enzymes developed by Thermo Fisher Scientific was used for the double-site digestion reaction. The cloning vector was constructed using the pEASY^®^-Blunt Simple kit. A 10 μL ligation system was prepared using the instructions (3 μL gel recovery product, 2 μL vector). After reacting at 37 °C for 25 min, the mixture was placed on ice or stored at −20 °C. Plasmids were extracted from the pRI101 and pTRV2 vector bacterial cultures after sequencing using the WholeForm Golden Kit and stored at −20 °C. A 20 μL double enzyme digestion system was prepared for the vector using Thermo Fisher FastDigest restriction enzymes. The mixture was incubated at 37 °C for 40 min. The products were validated using 1% agarose gel electrophoresis and recovered from the gel. A 10 μL homologous recombination system was prepared using the ClonExpress II kit. It was incubated at 37 °C for 30 min, placed on ice to terminate the reaction, and stored at −20 °C. The recombinant product was transformed into DH5α competent cells, followed by an ice bath, heat shock, and recovery steps. It was placed on a kanamycin-containing medium. Positive clones were selected for sequencing and plasmid extraction. Primers were used for PCR to amplify the target fragment and ligate it to the vector. Recombinant plasmids were obtained after transformation. They were screened, sequenced, and transformed into GV3101 and LBA4404 *Agrobacterium tumefaciens* competent cells. They were placed on a double-antibiotic medium after recovery. Positive clones were selected for verification using colony PCR and stored in glycerol at −80 °C [57,58].

Genetic Transformation and Functional Verification: The pTRV1 and pTRV2 bacterial suspensions were combined 1:1 and left in the dark for three to five hours after the bacteria were cultivated in LB medium containing antibiotics. They were shaken, diluted, and resuspended to OD600 = 0.6–1.5. Four to six true leaf seedlings were chosen for tobacco infection, and a needleless syringe was used to penetrate the abaxial side of the leaves. Following a 12 h dark incubation period, the seedlings were cultivated under light for three days. Samples were identified using qPCR. The functional leaves were immersed in the bacterial suspension with the abaxial side down and vacuum-infiltrated for 15 to 20 min to infect Gala tissue culture seedlings. After the excess suspension was removed, the plants were incubated in the dark for one day and under light for five to seven days. After removing the excess bacterial solution, the mixture was incubated for one day in the dark and five to seven days in the light. qPCR was used to determine phenotypic characteristics [59,60].

The seeds were sterilized with 75% ethanol and 40% NaClO, vernalized in the dark for two days at 4 °C, and planted on MS media free of antibiotics to infect them with *Arabidopsis*. They were grown for 7–10 days at 20–21 °C before being moved into pots with a 1:3 ratio of vermiculite to substrate. The soil was kept moist to encourage bolting. The plants were watered, and the siliques were trimmed during the bolting stage. After the bacterial solution was adjusted to an OD600 of 0.8, the inflorescences were submerged in the solution and dried, and the seeds were harvested. The second-generation seeds were planted on kanamycin-containing MS media to obtain resistant seedlings. We extracted RNA and performed cDNA reverse transcription and PCR identification to confirm the transformed lines (pre-denaturation at 94 °C for 3 min, 30 cycles of 94 °C for 30 s, 58 °C for 30 s, 72 °C for 1 min, and final extension at 72 °C for 5 min) [61,62,63].

qRT-PCR analysis: A fluorescent quantitative kit was used for amplification, with malondialdehyde as the reference gene. Pre-denaturation was performed at 95 °C for 30 s, followed by 40 cycles of denaturation at 95 °C for 5 s and annealing at 60 °C for 30 s. A 2^−ΔΔCT^ (ΔCT = CT, target − CT, reference and ΔΔCT = ΔCT, test − ΔCT) control was used to determine relative gene expression levels [64].

### 4.4. Data Statistics and Analysis

Excel 2021 and GraphPad Prism 8 were used to handle the data and create the charts. Using SPSS 29.0, one-way analysis of variance (ANOVA) and Duncan’s multiple range test were carried out; *p* < 0.05 was deemed statistically significant. “Mean ± standard deviation” is how the data are displayed [65].

## 5. Conclusions

1. Partial shading (30% to 35%) increased photosynthetic efficiency and chlorophyll content in ‘Duojiao’ crabapple leaves, improving their light resistance and enabling them to turn green earlier.
2. Chlorophyll synthesis and leaf color were regulated by the genes *MsCAO*, *MsCPOX*, and *MsGLK1*, with *MsCPOX* exhibiting the greatest positive effect on plant growth.

## Figures and Tables

**Table 1:** Key enzyme activity of chlorophyll metabolism in crabapple leaves.

Date	Dispose	Plant Glutamyl-tRNA Reductase	Plant δ-Aminolevulinic Acid Dehydratase	Plant Uroporphyrinogen III Synthase	Chlorophyllase	Plant Mg-Dechelatase
31 May	Dck	45.98 ± 0.5 c	392.66 ± 7.99 c	142.5 ± 3.6 c	171.48 ± 2.83 bc	2046.02 ± 26.08 c
	D1	45.64 ± 0.41 cd	392.4 ± 7.01 c	148.81 ± 2.26 b	167.14 ± 3.76 cd	2040.22 ± 3.24 c
	D2	44.85 ± 0.37 d	392.4 ± 6.99 c	150.23 ± 2.34 b	164.69 ± 3.71 de	1928.38 ± 34.26 d
	D4	43.57 ± 0.91 e	368.05 ± 7.52 d	150.8 ± 1.28 b	160.98 ± 3.72 e	1908.74 ± 33.37 d
	Xck	52.43 ± 0.63 a	417.26 ± 5.75 a	165.34 ± 3.03 a	175.81 ± 2.26 ab	2075.57 ± 31.2 bc
	X1	51.69 ± 0.49 b	414.63 ± 2.02 a	154.49 ± 1.13 b	176.01 ± 3.28 ab	2084.17 ± 30.02 bc
	X2	51.3 ± 1.08 b	408.55 ± 6.25 ab	153.59 ± 2.69 b	176.98 ± 4.85 ab	2111.29 ± 20.82 ab
	X4	50.61 ± 0.75 b	400.86 ± 6.65 bc	152.8 ± 3.16 b	180.08 ± 0.64 a	2154.12 ± 37.76 a
7 June	Dck	46.85 ± 0.57 b	393.01 ± 2.29 a	143.02 ± 1.09 de	174.61 ± 1.44 c	1963.32 ± 41.63 d
	D1	46.48 ± 0.26 bc	388.55 ± 4.92 ab	149.52 ± 3.86 c	162.65 ± 2.72 de	1761.36 ± 0.07 e
	D2	45.11 ± 0.84 cd	382.77 ± 4.08 bc	156.18 ± 2.95 b	159.67 ± 2.59 e	1750.73 ± 23.19 e
	D4	43.29 ± 1.02 e	347.38 ± 8.03 e	160.83 ± 0.78 a	158.69 ± 4.14 e	1649.7 ± 15.99 f
	Xck	50.06 ± 0.8 a	394.63 ± 4.09 a	163.33 ± 2.32 a	179.53 ± 0.71 bc	2072.25 ± 15.36 c
	X1	48.94 ± 0.97 a	375.7 ± 2.65 c	146.23 ± 0.37 cd	180.55 ± 2.11 bc	2111.07 ± 12.83 c
	X2	48.88 ± 0.73 a	363.57 ± 4.61 d	139.01 ± 2.41 e	182.86 ± 5.14 ab	2259.25 ± 54.1 b
	X4	43.82 ± 1.09 de	348.53 ± 2.46 e	137.65 ± 3.9 e	186.55 ± 0.68 a	2319.97 ± 14.87 a
14 June	Dck	48.38 ± 0.13 b	398.7 ± 4.17 b	148.52 ± 0.19 c	173.51 ± 0.62 c	1876.29 ± 4.21 d
	D1	40.25 ± 0.62 d	382.58 ± 7.3 c	163.56 ± 1.21 b	161.67 ± 1.63 d	1855.81 ± 39.31 de
	D2	38.62 ± 0.38 e	373.99 ± 4.53 cd	165.86 ± 1.33 b	160.07 ± 2.55 d	1813.23 ± 38.72 ef
	D4	35.63 ± 0.74 f	326.02 ± 7.9 f	169.1 ± 2.44 a	157.63 ± 0.8 e	1775.21 ± 16.43 f
	Xck	50.25 ± 0.06 a	400.45 ± 5.2 a	164.86 ± 1.24 b	180.48 ± 2.63 b	2273.12 ± 12.23 c
	X1	47.71 ± 1.14 c	372.98 ± 3.69 d	143.32 ± 1.43 d	183.48 ± 2.98 b	2297.25 ± 32.13 c
	X2	47.38 ± 0.18 c	346.24 ± 4.05 e	141.39 ± 1.28 d	210.22 ± 3.06 a	2436.52 ± 16.67 b
	X4	39.12 ± 0.8 e	323.93 ± 1.45 f	138.62 ± 0.87 e	212.74 ± 2.95 a	2505.28 ± 40.99 a
21 June	Dck	49.55 ± 0.55 a	397.38 ± 8.19 a	161.65 ± 2.04 ab	172.24 ± 4.04 cd	2231.36 ± 36.65 c
	D1	37.99 ± 0.81 c	380.33 ± 4.19 bc	134.06 ± 1.4 d	160.09 ± 4.24 de	2173.75 ± 35.54 d
	D2	35.1 ± 0.4 e	369.6 ± 6.3 c	138.44 ± 0.8 c	158.58 ± 4.03 ef	2085.48 ± 17.17 e
	D4	31.9 ± 0.19 f	323.73 ± 6.11 e	164.51 ± 0.98 a	155.66 ± 2.13 f	2025.07 ± 34.89 f
	Xck	50.66 ± 1.02 a	381.35 ± 4.19 b	159.37 ± 0.8 b	175.71 ± 1.08 c	2380.04 ± 16.67 b
	X1	45.8 ± 0.51 b	340.44 ± 6.23 d	135.48 ± 2.84 cd	190.87 ± 1.62 b	2425.3 ± 31.73 b
	X2	43.65 ± 1.13 c	327.8 ± 5.1 e	126.37 ± 3.36 e	220.31 ± 1.79 a	2493.38 ± 32.58 a
	X4	36.98 ± 0.38 d	318.73 ± 3.22 f	121.02 ± 0.93 f	223.97 ± 2.32 a	2526.67 ± 20.77 a

Note: Values in the same column with different small letters indicate a significant difference (*p* < 0.05).

## Data Availability

Data is contained within the article.

## Supplementary Materials

The following supporting information can be downloaded at: https://www.mdpi.com/article/10.3390/plants15101552/s1, Figures S1–S6: Sequence comparison of key candidate genes and cloned genes for leaf color in ‘Duojiao’ crabapple. Figure S7: ‘Duojiao’ crabapple leaf color key candidate gene phylogenetic tree; Figure S8: ‘Duojiao’ crabapple leaf color key candidate gene protein secondary structure peak diagram; Figure S9: Tertiary structure models of key candidate gene proteins for ‘Duojiao’ crabapple leaf color; Table S1: Analysis of the physicochemical properties of key candidate gene proteins for the leaf color of ‘Duojiao’ crabapple.

## Appendix A

**Table A1:** Gene primer.

Primer Used in This Study	Primer Sequence (5′-3′)	Purpose
MsCAO-pRI101-F	ttgatacatatgcccgtcgacATGAGTCTGTCAGCTTCAAACGC	gene cloning
MsCAO-pRI101-R	tcagaattcggtacccccgggAGTTGATTTGGTGAAGGGTAGTTGT	
MsCPOX-pRI101-F	GttgatacatatgcccgtcgacATGGCGACTGCAGTGCTACC	gene cloning
MsCPOX-pRI101-R	tcagaattcggtacccccgggCACAAGATGTTTCACCATGTAAGGT	
MsGLK1-pRI101-F	ttgatacatatgcccgtcgacATGCTTATTTTATCACCTTTGCGG	gene cloning
MsGLK1-pRI101-R	tcagaattcggtacccccgggGGCACAGGAGAGTGGTATTTTTG	
MsCAO-pTRV2-F	taaggttaccgaattcTGATGTGGAGGATCCGAGGT	gene cloning
MsCAO-pTRV2-R	agacgcgtgagctcggtaccTCCACGAAAGAGAACCCACG	
MsCPOX-pTRV2-F	taaggttaccgaattcTCCGAGCCATAGCCGAAATC	gene cloning
MsCPOX-pTRV2-R	agacgcgtgagctcggtaccACGCAAATATTCCCGGTGGT	
MsGLK1-pTRV2-F	taaggttaccgaattcTTTCCGGGGGAATGGGAATG	gene cloning
MsGLK1-pTRV2-R	AgacgcgtgagctcggtaccAGCTGCTGCCCCATACATTT	
RT-MsCAO-F	GCGGCGGGAACTGTTTATTG	semi-quantitative/ qRT-PCR
RT-MsCAO-R	ACAACATGTAATTGGGTGGTGGT	
RT-MsCPOX-F	CGGATGGAAGACTTGGGCTT	semi-quantitative/ qRT-PCR
RT-MsCPOX-R	AGCACTTCCCCATGAGTTCG	
RT-GLK1-F	CTCCTGTGCCTGATGGTGT	semi-quantitative/ qRT-PCR
RT-GLK1-R	AGGCAGAAGAATGACGGGC	
RT-MdActin-F	TGACCGAATGAGCAAGGAAATTACT	semi-quantitative/ qRT-PCR
RT-MdActin-R	TACTCAGCTTTGGCAATCCACATC	
