# Supplementary material for: Research on the Physiological Response Mechanism and Expression of Key Leaf Color Genes in ‘Duojiao’ Crabapple Under Partial Shading
Source: Plants (Basel). 2026 May 19;15(10):1552. doi: 10.3390/plants15101552 (PMC13210476; doi:10.3390/plants15101552)
Supplement: Supplementary file 1 [file plants-15-01552-s001.zip › plants-4227188-supplementary.pdf]

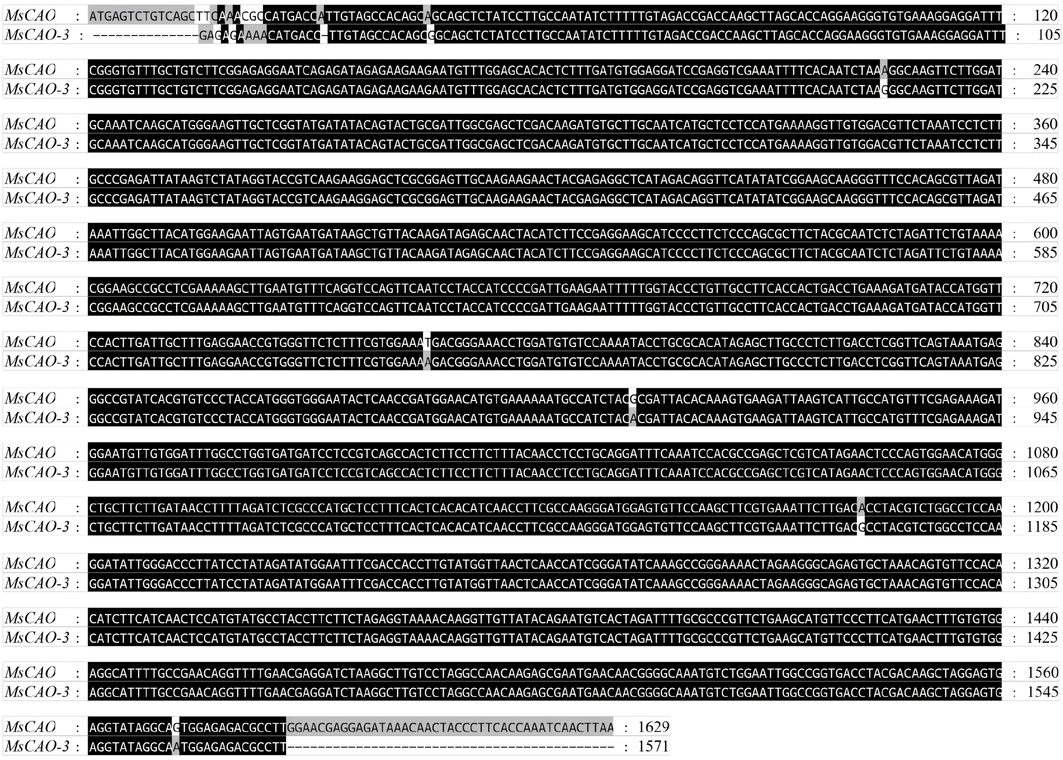

Figure S1: Figure S1 Sequence alignment of the key candidate gene *MsCAO* for leaf color in crabapple 'Duojiao' and the cloned gene *MsCAO-3*.

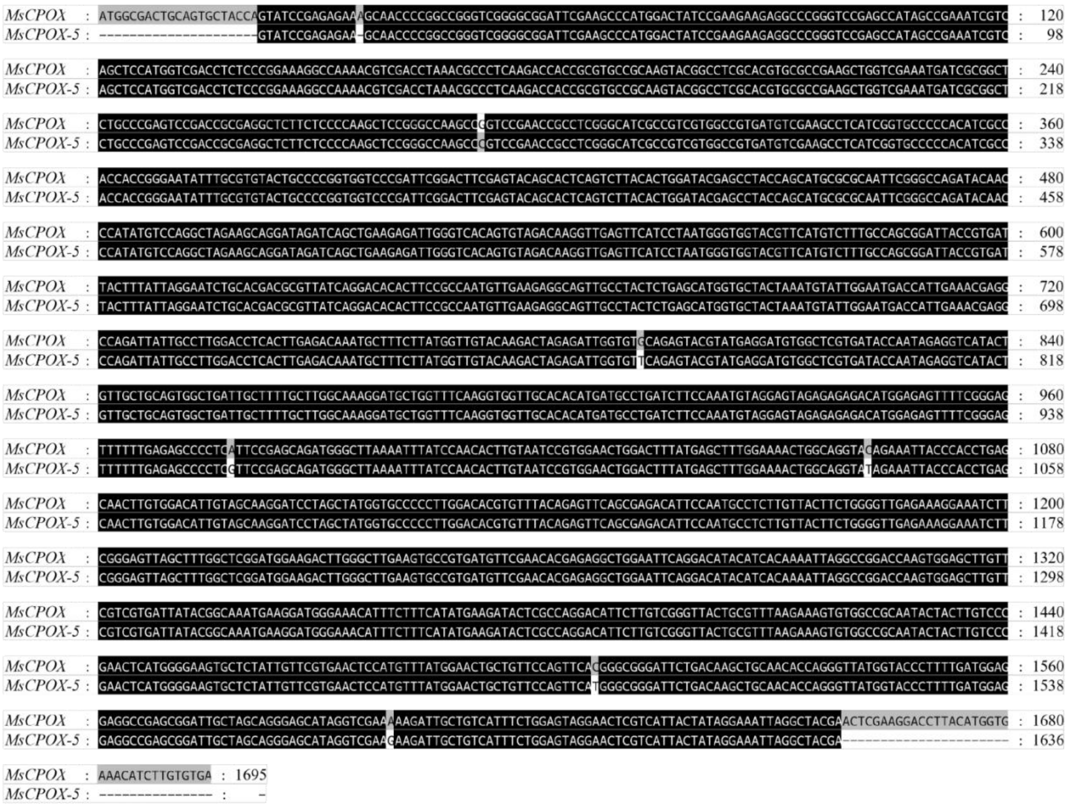

Figure S2: Sequence alignment of the key candidate gene *MsCPOX* for leaf color in crabapple 'Duojiao' and the cloned gene *MsCPOX-5*.

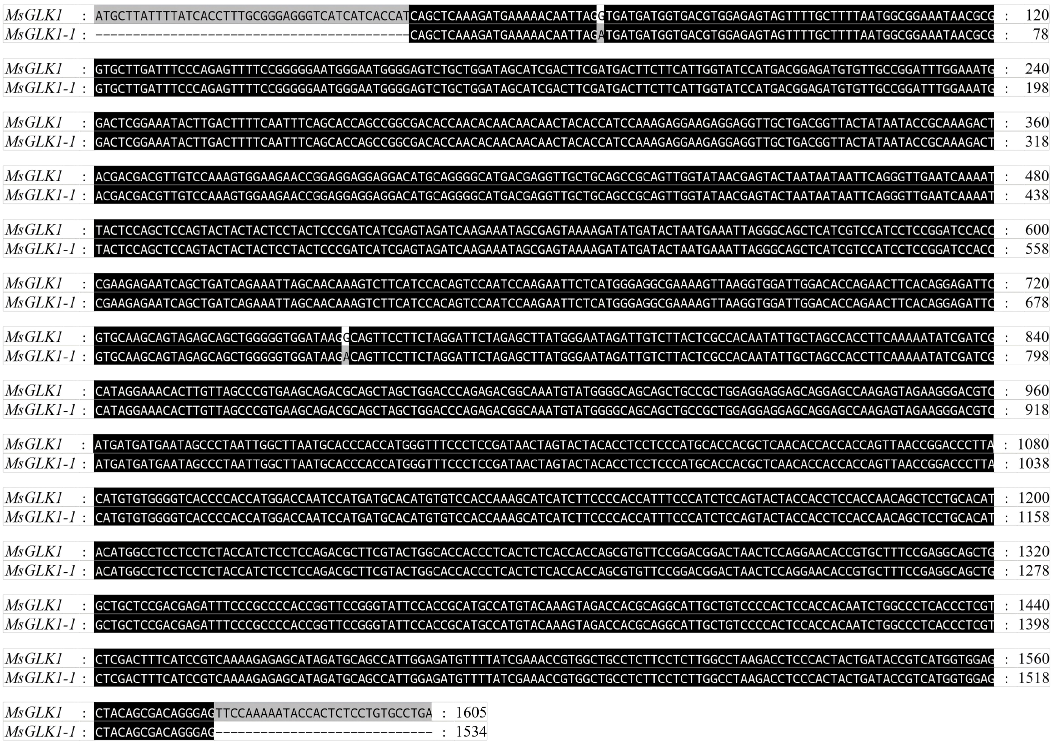

Figure S3: Sequence alignment of the key candidate gene *MsGLK1* for leaf color in crabapple 'Duojiao' and the cloned gene *MsGLK1-1.*

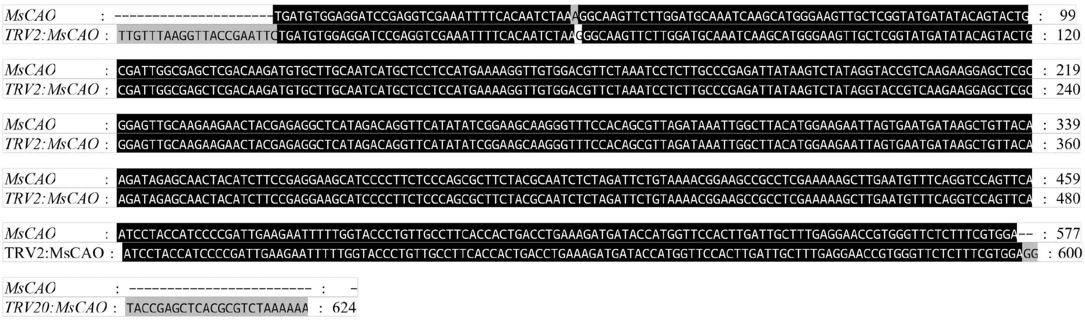

Figure S4: Sequence alignment of the key candidate gene *MsCAO* for leaf color in crabapple 'Duojiao' and the cloned gene *TRV2:MsCAO.*

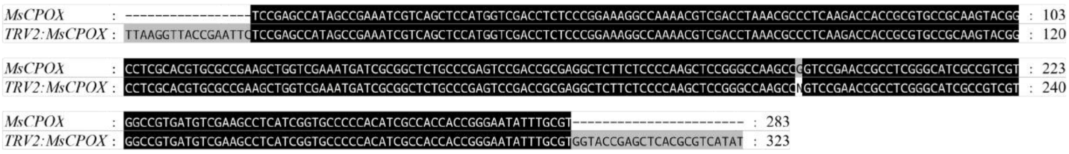

Figure S5: Sequence alignment of the key candidate gene *MsCPOX* for leaf color in crabapple 'Duojiao' and the cloned gene *TRV2:MsCPOX.*

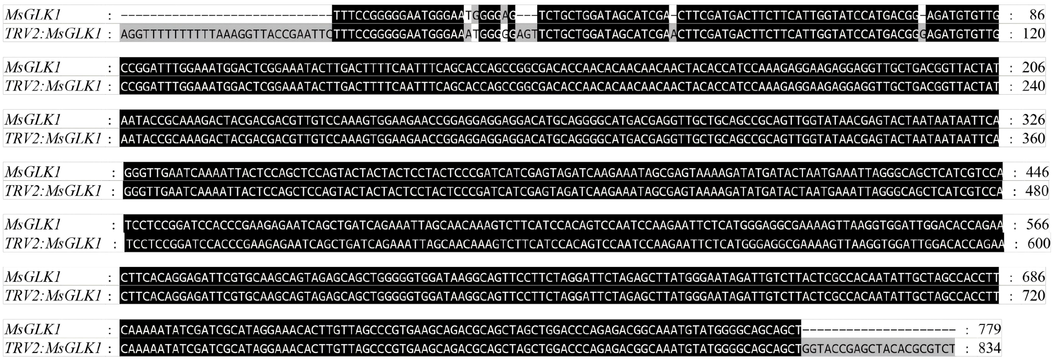

Figure S6: Sequence alignment of the key candidate gene *MsGLK1* for leaf color in crabapple 'Duojiao' and the cloned gene *TRV2:MsGLK1*.

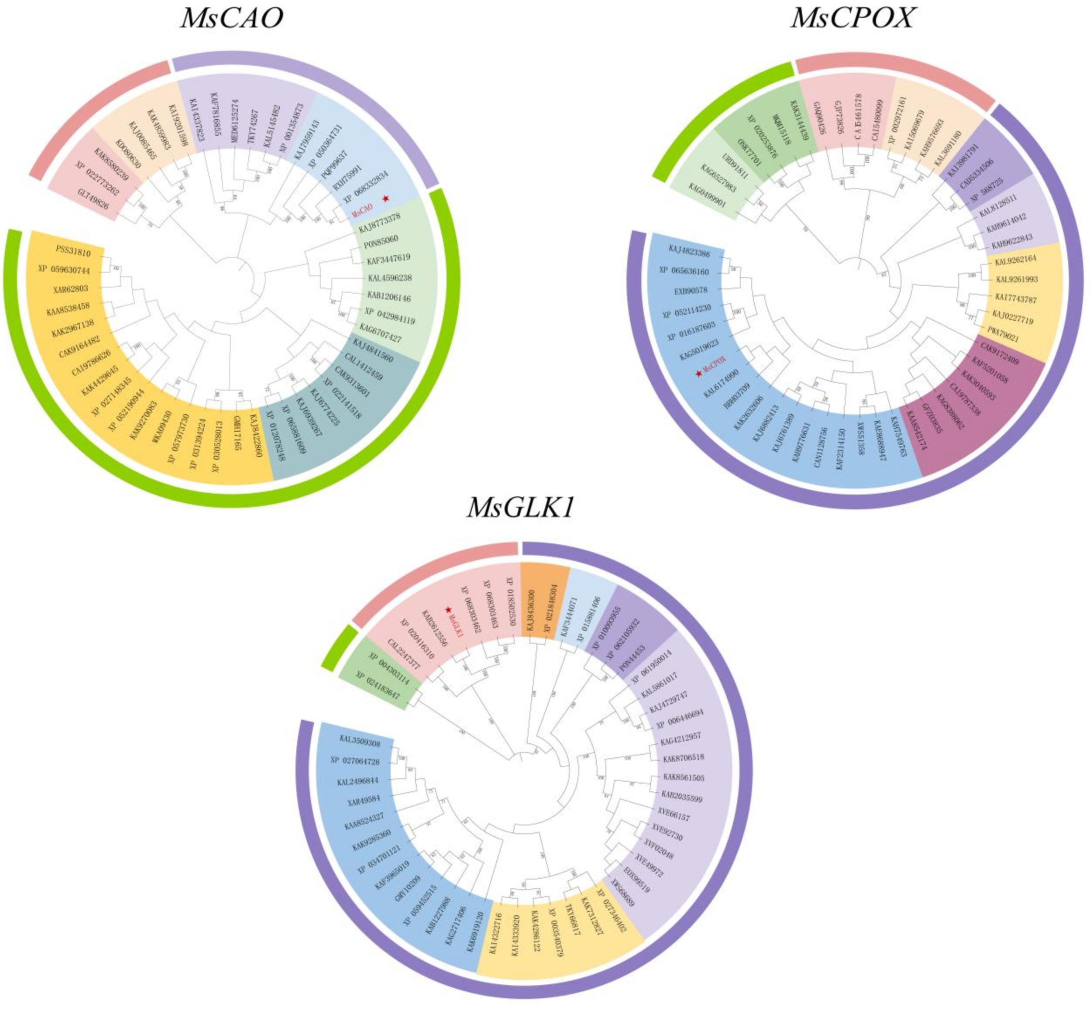

Figure S7: 'Duojiao' crabapple Leaf Color Key Candidate Gene Phylogenetic Tree

Table S1: Analysis of the Physicochemical Properties of Key Candidate Gene Proteins for the Leaf Color of crabapple 'Duojiao'

Gene ID	Gene name	bp	Relative molecular mass of protein	Relative molecula r mass of protein	Instability coefficient	Average hydrophobicity
LOC103431681	MsCAO	1629	61306.18	8.15	44.10	-0.393
LOC103408418	MsCPOX	1695	63420.81	8.87	34.32	-0.364
LOC103444434	MsGLK1	1605	58517.54	6.19	53.29	-0.637

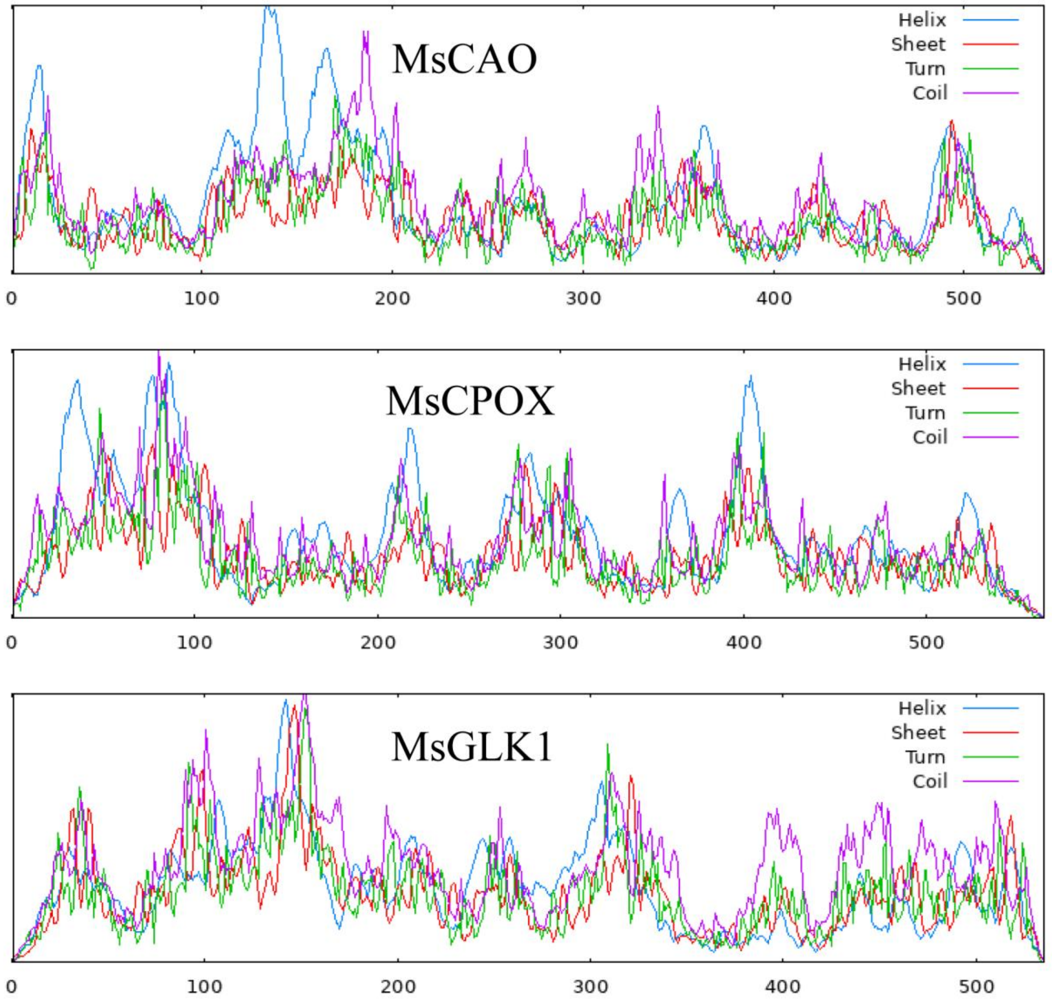

Figure S8: 'Duojiao' crabapple leaf color key candidate gene protein secondary structure peak diagram

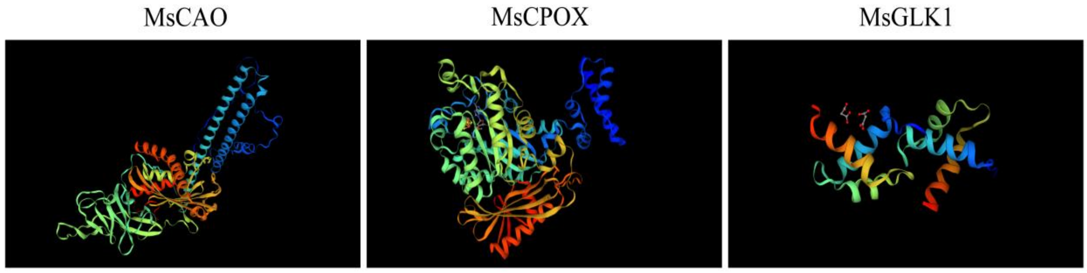

Figure S9: Tertiary structure models of key candidate gene proteins for 'Duojiao' crabapple leaf color
